# Is There a Vegetable Pepsin?

**Published:** 1892-09

**Authors:** 


### Selected Articles.

### IS THERE A VEGETABLE PEPSIN?

It is said that in Africa certain tribes render their beefsteaks tender by rubbing them with the juice of a species of the paw paw,—a native plant. There is a preparation now in extensive and very popular use called Papoid, a scientifically prepared extract containing this solvent principle, for which are claimed very extraordinary properties as a digestive agent. Dr. Frank Woodbury, in a lengthy article in the *New York Medical Journal* of July 30th, details an extensive experience with this agent, and gives the result of his observations. Should these results be verified by further experience, and by other observers, it would seem to furnish conclusive evidence that a vegetable pepsin has really been discovered. Dr. Woodbury says:

During the past year, having devoted considerable attention to the clinical application of Papoid, especially in digestive disorders, I have had the satisfaction of witnessing a number of very interesting results, to which I wish briefly to direct attention. The successful application of physiological data must be my excuse for again directing attention to a remedy which has been studied by such eminent investigators as Wurtz, Bouchut, Finckler, Rossbach, Roy and Wittmach, and one, furthermore, the physiological and therapeutical actions of which may be regarded as pretty fully established. I point out very briefly some of the clinical uses and the conditions of its successful employment.

The physiological actions of Papoid as a digestive agent, have been thoroughly established. It acts upon albuminoids, hydrating and converting them into peptones, as fully demonstrated by Herschell. It converts starch with great promptness, the ultimate product being maltose. It emulsifies fat. Herschell declares that it has a direct tonic action on the stomach, stimulates the secretion of gastric juice or pepsinogen. It acts at all temperatures, but attains its maxim activity at about 130° F. In several important points, it differs from pepsin. Papoid acts best in an alkaline solution, but also can act in fluids with an acid or neutral reaction; pepsin requires an acid solution. Papoid is freely soluble, and is most active when in concentrated form; pepsin requires free dilution. Herschell also points out the greater digestive power possessed by Papoid than either pepsin or pancreatine, and states "that it can be used when pepsin is contra-indicated or powerless." Papoid has no action upon living tissues, and is positively innocuous when swallowed in any quantity that is likely to be administered. Papoid is useful when digestion has been overtaxed, or when the secretion of gastric juice is absent or deficient. Experiments of my own and others, have satisfied my mind of the remarkable digestive activity of Papoid. In one of the experiments referred to, the constituents of a hearty dinner of bread, meat, potatoes, peas, mince-pie, and other substantials, were placed in a test tube and treated with papoid and bicarbonate of sodium, and a small amount of water. The result was very satisfactory indeed; the meat rapidly softened, and the other ingredients gradually disintegrated, forming a pultaceous mass, which finally separated into a grumous sediment and an overlying albuminous, dark-colored liquid. Papoid acts in an alkaline solution even better than in acid media. It is evident that it is specially useful where there is indigestion due to deficient secretion of gastric juice. In such cases, the administration of an alkaline solution of Papoid favors gastric digestion both directly and indirectly: First, by digesting albuminates and softening masses of food; secondly, by the action of the papoid in stimulating the secretion of pepsin gland. It retards the fermentation of the undigested masses of food in the stomach, and prepares them for intestinal digestion. In the contrary case, where the stomach contents poured into the duodenum are so acid that they prevent the action of the trypsin, Papoid prevents duodenal indigestion by taking the place of the pancreatic ferment. As Herschell says, it is obviously of no use to give pancreatine by the mouth, as it is at once destroyed by the acid of the stomach, and it is practically impossible to administer sufficient alkali to neutralize the excess of acid, and it would, moreover, be unwise, because it would stimulate still further the secretion of the acid. Papoid is of the greatest use here. In gastralgia, which often accompanies the condition just named, Papoid with bicarbonate of sodium gives immediate relief. It is also useful in irritable stomach, nausea and vomiting. In gastric catarrh, and the catarrhal conditions of the intestinal tract popularly known as biliousness, Papoid administered in hot water fifteen minutes before meals, cleanses off the mucus and places the mucous coat of the digestive organs in a good condition for secretion. It is useful in the treatment of irritative diarrhœa in young children, to whom it may be given in combination with salol or salicylate of bismuth. The use of papoid in treating disorders of the digestive organs may be summarized somewhat as follows:

1. In actual or relative deficiency of the gastric juice, or its constituents.

(a) Diminished secretion of gastric juice as a whole. Apepsia; anæmia and deficient blood supply, wasting diseases.

(b) Diminished proportion of pepsin; atonic dyspepsia, atrophy of the gastric troubles.

(c) Diminution of hydrochloric acid. Achlorhydria; Carcinoma.

(d) Relative deficiency of gastric juice, overfeeding.

2. In gastric catarrh.

(a) Where there is a tenacious mucus to be removed, thus enabling the food to come in contact with the mucous membrane.

(b) Where there is impaired digestion.

3. In excessive secretion of acid. To prevent duodenal dyspepsia.

4. In gastralgia, irritable stomach, nausea and vomiting.

5. Intestinal disorders.

(a) In constipation due to indigestion.

(b) In diarrhœa, as a sedative.

(c) In intestinal worms. (This claim the writer has not personally verified, but as the intestinal mucus, which shields the worms is removed by Papoid, it is easily understood that their removal would naturally result after its administration.)

6. In infectious disorders of the intestinal tract.

(a) Where there is abnormal fermentation by its antiseptic action, which may be heightened by combination.

(b) Where there are foreign substances present, its detergent effect may be utilized by cleaning out the debris from the intestinal contents by digestion.

7. In infantile indigestion; here Papoid not only readily peptonizes cow's milk, but the resulting curds are also soft and floculent, resembling those of breast milk.

The dose of Papoid ordinarilly, is one or two grains, but five grains or more may be used, the only objection being that of useless expense and waste, except where very prompt effects are desired.

Dr. A. Rose, of Lebanon, Ky., in the *Medical Mirror* recommonds very highly the use of GARDNER'S SYRUP OF HYDRIODIC ACID in numerous diseases, especially in syphillis, and says: "The anæmia disappeared, the continuous head-ache instantly ceased, sore throat got well at once (with a few applications of zinch chlor. gr. xl ad aquæ i ounce) and all glandular swelling disappeared. The gummata everywhere vanished into thin air, and the eruption was held in *statu quo* until I could give the patient a few calomel vapor baths, preceded by steam sweating." "But there is a wide field for the usefulness of this preparation that has little been dreamed of," says Dr. Rose, "viz.: in tubal disease of ovary, rheumatism of the uterus, leucorrhæa and membranous dysmenorrhœa."

---

The cuteness of the American in useful "notions" has been run pretty close by a Dutch apothecary, who has taken out a patent for an automatic doctoring machine. The machine is shaped like a man, but this signifies nothing beyond advertisement. It is divided into compartments, each bearing the label of some disease above the "slit;" a sufferer chooses that which refers to his complaint, drops in his money, and receives a pill, a powder, or a draught; suitable to his case. It wouldn't do to put these machines about Sydney or Brisbane streets after 11 p. m., as fellows who found all the hotels closed would probably drop coins in all the slots in their attempt to get a nip.—*Phar. Journal of Australasia, Sydney, N. S. W.*

